# A diffusion-based framework for designing molecules in flexible protein pockets

**DOI:** 10.1126/sciadv.aeb7045

**Published:** 2026-04-08

**Authors:** Jian Wang, Dong Yan Zhang, Shreshty Budakoti, Nikolay V. Dokholyan

**Affiliations:** ^1^ Department of Neurology and Neuroscience, University of Virginia, School of Medicine, Charlottesville, VA, USA.; ^2^ Eberly College of Science, Pennsylvania State University, University Park, PA, USA.; ^3^ Department of Biomedical Engineering, University of Virginia, School of Medicine, Charlottesville, VA, USA.

## Abstract

Designing molecules for flexible protein pockets poses a substantial challenge in structure-based drug discovery, as proteins often undergo conformational changes upon ligand binding. While deep learning–based methods have shown promise in molecular generation, they typically treat protein pockets as rigid structures, limiting their ability to capture the dynamic nature of protein-ligand interactions. Here, we present YuelDesign, a diffusion-based framework that jointly models the pocket structures and ligand conformations of protein-ligand complexes. YuelDesign uses E3former to maintain rotational and translational equivariance. The framework incorporates dual diffusion processes, an elucidated diffusion model (EDM) for coordinates and a discrete denoising diffusion probabilistic model (D3PM) for ligand atom types, enabling iterative refinement of both geometry and chemical identity. Our results demonstrate that YuelDesign generates molecules with favorable drug-likeness, low synthetic complexity, diverse chemical functional groups, and docking energies comparable to native ligands. YuelDesign presents a versatile framework for designing drugs in flexible protein pockets, with promising implications for drug discovery applications.

## Teaser

YuelDesign generates drug-like molecules within flexible protein pockets using dual-diffusion models.

## INTRODUCTION

The design of three-dimensional (3D) molecular structures with specific properties remains a fundamental challenge in drug discovery. In recent years, generative artificial intelligence techniques, especially autoregressive models (*1*) and diffusion models (*2*), have transformed de novo drug discovery (*3*) by facilitating the efficient generation of molecular structures. These generative approaches are broadly categorized into two main categories: 2D molecular topology generation, which involves constructing molecular graphs or string-based representations such as SMILES (*4*) and SELFIES (*5*), and 3D molecular conformation generation, which aims to predict atomic positions in three-dimensional space.

In the domain of 2D molecular generation, commonly used approaches include autoregressive models (*6*–*8*) [e.g., Recurrent Neural Networks (RNNs) for SMILES/SELFIES generation], variational autoencoders (VAEs) (*9*) [GraphVAE (*10*) and CGVAE (*11*)], generative adversarial networks (GAN)–based models such as MOLGAN (*12*), and flow-based frameworks including GraphNVP (*13*) and MoFlow (*14*). Concurrently, 3D molecular generation has made notable progress through a range of innovative approaches. For example, DiffSBDD (*15*) showcased the use of diffusion models in structure-based drug design by leveraging equivariant graph neural networks (*16*) to generate candidate molecules within protein binding pockets. Other notable diffusion-based methods include Pocket based Molecular Diffusion Model (PMDM) (*17*), DiffSMol (*18*), DiffBP (*19*), and TargetDiff (*20*).

A critical yet underexplored challenge in structure-based molecular design is the treatment of protein binding pockets during the generation process. This challenge is analogous to the distinction between rigid and flexible docking (*21*, *22*) in protein-ligand docking studies. While rigid docking simplifies the binding site as a static structure, flexible docking allows for conformational adjustments, enabling more accurate predictions of binding modes and affinities. This concept, known as induced fit (*23*, *24*), underscores how ligand binding can trigger structural rearrangements within the protein pocket, potentially exposing or altering key interaction sites. Induced fit also substantially affects other calculations, such as binding affinity prediction (*25*, *26*), virtual screening (*27*), and target identification (*28*). Despite its relevance, the dynamic nature of protein pockets remains largely overlooked in current molecular design frameworks. Approaches such as DiffSBDD typically treat the binding pocket as a rigid entity.

**Fig. 1.**
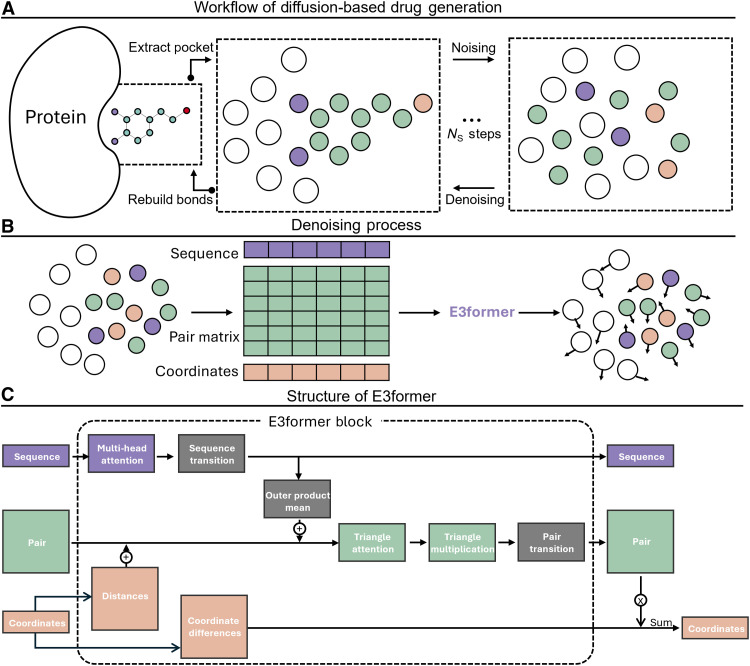
Workflow and architecture of YuelDesign. (**A**) Pipeline begins with protein pocket extraction, followed by iterative noising (*N*_s_ steps) and denoising to generate 3D molecular conformations. Bond reconstruction is applied postdenoising. *N*_s_, number of steps. (**B**) Denoising via E3former. Atom types are encoded into sequence features. Atom-atom interactions are encoded into pair features. (**C**) E3former architecture. Within each E3former block, sequence attention, sequence transition, and sequence outer product modules propagate information among atoms. Pair features are updated through triangle attention and triangle multiplication modules. E3former also includes an equivariant coordinate head that converts the final pair embeddings into coordinate displacements. This module aggregates weighted direction vectors between atoms, ensuring that the predicted updates are equivariant to rotations and translations.

In this work, we present YuelDesign, a diffusion-based molecular design framework (Fig. 1) specifically developed to address the challenge of designing molecules for flexible protein pockets. Our framework integrates several key innovations to tackle this complexity. First, we use a full-atom representation that simultaneously generates both the protein pocket structure and the small-molecule structure during the diffusion process. This joint generation approach explicitly models protein flexibility, allowing the protein structure to evolve alongside the ligand throughout the generation process. Second, we introduce an architecture called E3former, which serves as the backbone network in our diffusion framework. The E3former is adapted from AlphaFold (*29*) Evoformer architecture, incorporating triangular attention mechanisms while directly outputting coordinates in an E (3)–equivariant manner (unlike Evoformer which outputs distograms). E3former enables our model to handle the increased complexity of full-atom representations, where the total atom count can reach up to 400 atoms (including protein side chains) compared to the around 15 to 50 atoms for ligands alone in previous approaches. Third, we adopt a hybrid diffusion strategy: We use D3PM (denoising diffusion probabilistic models for discrete data) (*30*) to handle atom type prediction (discrete variables) while using EDM (*31*) for generating continuous coordinates. The E3former architecture simultaneously predicts both atom types (using D3PM) and coordinates (using EDM), respecting the discrete nature of atom types while maintaining equivariance for coordinate generation.

We conducted extensive evaluations to demonstrate the effectiveness of YuelDesign across multiple dimensions. First, we assessed the chemical properties of the generated molecules, showing high drug likeness and low synthetic complexity. Second, we analyzed the chemical diversity of generated molecules. Third, we evaluated the generated pocket conformation and protein-ligand interactions, and we found YuelDesign recapitulated pocket conformation and produced new residue-ligand contacts. Last, we performed detailed analyses of the denoising process to understand how the model systematically refines molecular structures through atom type transitions, bond dynamics, and conformational changes.

## RESULTS

### Chemical property analysis of diffusion-generated molecules

**Fig. 2.**
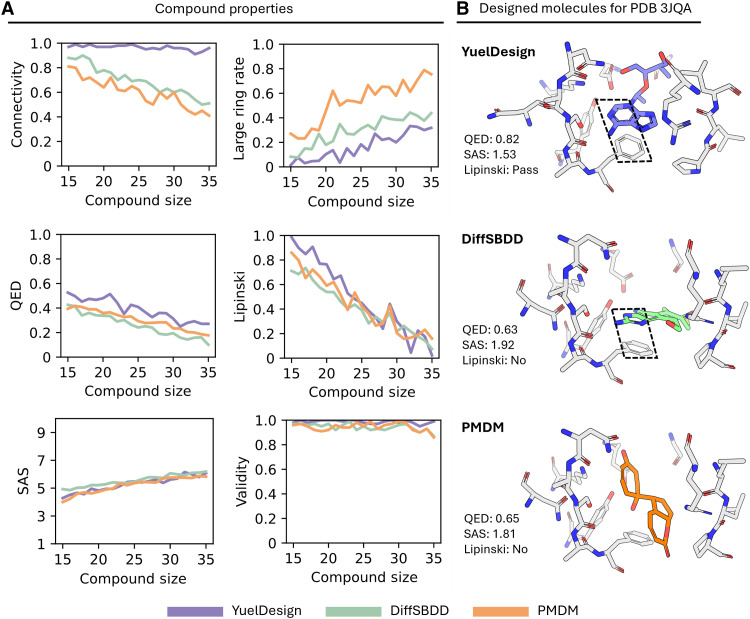
Evaluation of generative molecules through multiple metrics. (**A**) Evaluation of connectivity, macrocycle (more than 6 atoms) formation rate, QED, Lipinski’s RO5 compliance (pass/fail ratios), SAS, and validity. Compound size is the number of heavy atoms. (**B**) Comparison of molecules designed by YuelDesign, DiffSBDD, and PMDM for Pteridine reductase 1 (PDB ID: 3JQA). Inside the black box is the π-π stacking interaction.

We conducted a comprehensive evaluation of the chemical properties of generated molecules, comparing YuelDesign with DiffSBDD and PMDM across multiple metrics (Figure 2). The analysis includes quantitative estimate of drug-likeness (QED) (*32*), Lipinski’s Rule of Five (RO5) compliance, synthetic accessibility score (SAS) (*33*), large ring formation rate, chemical validity, and connectivity.

We first evaluated connectivity and the large-ring ratio (Fig. 2A). Connectivity, whether all atoms in a molecule form a single bonded component, remains a common limitation in diffusion-based molecular generation, as these models predict atomic coordinates independently without explicitly enforcing chemical bonding constraints, often resulting in fragmented structures. Large rings, defined as cycles containing more than six atoms, are also of interest because they pose challenges for chemical synthesis and can negatively affect drug-like properties. YueIDesign maintains relatively high connectivity across all molecule sizes, while PMDM and DiffSBDD decline noticeably with increasing size. Molecule size is defined as the number of heavy atoms in the compound. For large rings (cycles >6 atoms), PMDM exhibits a substantially higher large-ring ratio (especially for larger molecules), while DiffSBDD maintains a moderate rate, and YueIDesign shows the lowest large-ring rate across all molecule sizes (Fig. 2A). Given that excessive large rings are unfavorable (as they typically increase synthetic difficulty and impair drug-like properties), this hierarchy (YueIDesign < DiffSBDD < PMDM in large-ring ratio) reflects the advantage of YuelDesign in avoiding problematic structural motifs. The increasing trend also suggests that controlling ring formation becomes increasingly challenging as molecular size rises.

We next assessed the drug-likeness of the generated molecules using two widely adopted metrics: QED, a composite score ranging from 0 to 1 that integrates multiple molecular descriptors, and Lipinski’s RO5, which evaluates four key physicochemical properties relevant to oral bioavailability. For QED, all three models show a downward trend in QED as molecular size increases (Fig. 2A), and YueIDesign consistently maintains a higher QED than DiffSBDD and PMDM across all size ranges. For Lipinski’s RO5 compliance, YueIDesign outperforms both DiffSBDD and PMDM for small molecules; as molecular size grows, all three models exhibit notable declines in RO5 compliance. This trend aligns with the inherent design of Lipinski’s rules: Since the RO5 evaluates physicochemical properties (e.g., molecular weight) that scale with atom count, larger molecules naturally have a higher likelihood of violating these criteria.

We also evaluated the synthetic accessibility of the generated molecules using the SAS score (Materials and Methods), which ranges from 1 (easy to synthesize) to 10 (very difficult). This metric accounts for molecular complexity, the presence of uncommon structural motifs, and similarity to known compounds. As shown in Fig. 2A, the SAS values of molecules generated by all three methods are relatively close across different molecular sizes. Last, we evaluated molecular validity, a fundamental measure of chemical feasibility that ensures generated structures comply with basic chemical rules and constraints. Validity was assessed using MolVS (*34*), which has a built-in validator to flag potentially problematic molecular features (see Materials and Methods). As shown in Fig. 2A, all three methods maintains a validity rate near 1.0 across all molecular size ranges, except that PMDM has a decline for molecular size of 35. For property analyses that require sanitized molecules (QED, Lipinski, SAS, etc.), we use only valid and connected molecules.

The superior performance of YuelDesign may be attributed to its ability to account for receptor pocket flexibility; for example, as shown in Fig. 2B, YuelDesign generates molecules with higher QED and lower SAS scores while simultaneously inducing conformational changes in the pocket.

### Molecular diversity assessment of diffusion model outputs

**Fig. 3.**
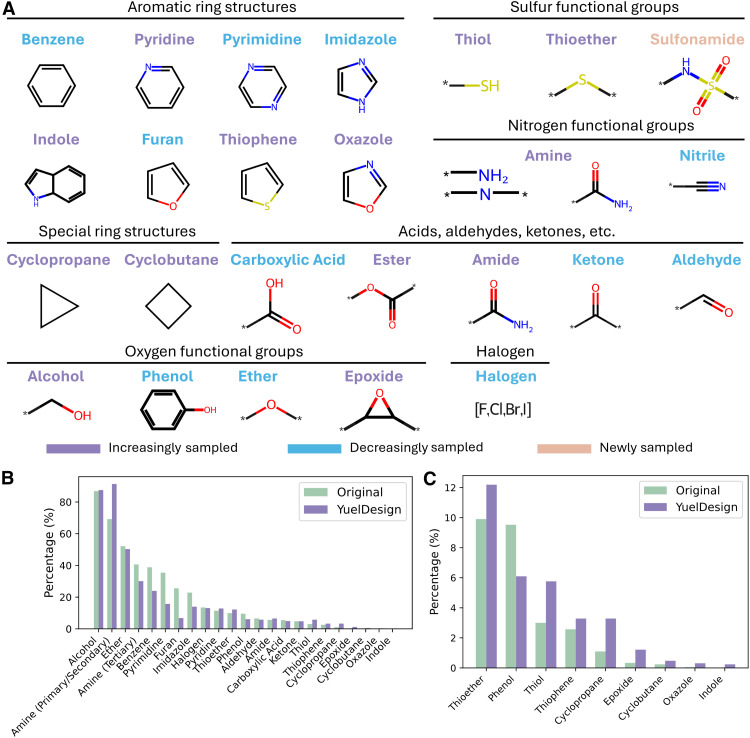
Functional group diversity analysis. (**A**) Representative structures of organic functional groups evaluated, categorized by chemical class (aromatic rings, N/S/O-containing groups, halogens, etc.). (**B** and **C**) Bar plots comparing the percentage distribution of functional groups in native ligands (green) versus YuelDesign (blue).

We conducted a thorough analysis of chemical diversity by examining the frequency distribution of functional groups in both generated molecules and native ligands (Fig. 3A). This analysis includes various structural motifs, such as aromatic rings, specialized ring systems, and functional groups that contain sulfur, nitrogen, and oxygen atoms. In addition, we assessed the presence of common organic functional groups such as acids, aldehydes, ketones, esters, amides, and other specialized moieties.

The results revealed overall similarity between generated molecules and native ligands in terms of functional group distributions (Fig. 3B). Alcohol groups were among the most prevalent in both sets, present in ~85% of molecules, while amine groups ranked second in native ligands and first in generated molecules, occurring in ~70% and ~90% of molecules, respectively. A difference worth noting was the occurrence of small rings, such as cyclopropane, cyclobutene, and epoxide (Fig. 3C). Although their frequency remains low, these motifs were slightly enriched in generated molecules. Small rings are generally avoided in drug-like compounds due to potential synthetic difficulty and strain-related instability. This tendency implies a limitation of diffusion-based models: During generation from Gaussian noise, three- or four-atom rings can form easily, as they do not inherently violate bond lengths or angles, leading to the overproduction of these strained motifs.

### Receptor and protein-ligand interaction analysis

**Fig. 4.**
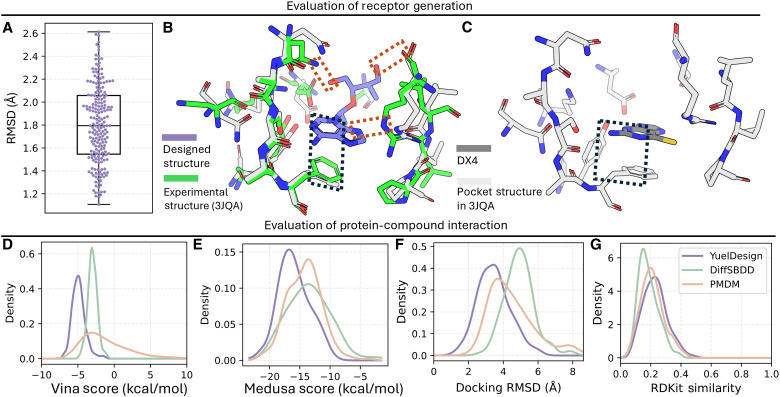
Evaluation of receptor pocket generation and protein-compound interaction through multiple metrics. (**A**) Distribution of RMSD of generated pocket structure. (**B**) Alignment of the generated pocket structure of 3JQA and the experimental structure of 3JQA. Inside the black dashed box is the π–π interaction; inside the orange boxes are polar contacts. (**C**) Experimental structure of 3JQA/DX4 complex. DX4 (2-amino-1,9-dihydro-6H-purine-6-thione) is the native ligand of 3JQA. (**D**) Distribution of Autodock Vina score (kcal/mol) of all the generated molecules binding with the pocket. (**E**) Distribution of Medusa score (kcal/mol) of all the generated molecules binding with the pocket. (**F**) Distribution of RMSD between generated and redocking poses with MedusaDock. (**G**) Distribution of the RDKit Daylight-like fingerprints Tanimoto score between the generated molecules and the original molecule (DX4).

Because YuelDesign can simultaneously generate protein pockets and small-molecule structures, we evaluated the structural quality of the generated protein binding pockets. Comparing the generated pockets with their native counterparts using a root mean square deviation (RMSD), we observed a median RMSD of 1.8 Å (Fig. 4A), which is reasonable, as excessively high RMSD would indicate inaccurate side-chain conformations. In reality, protein-ligand binding generally induces only minor conformational adjustments, mainly through side-chain rotations to optimize interactions with the ligand. Traditional flexible docking methods, such as MedusaDock (*21*, *22*) and AutoDock Vina (*35*), also rely on protein side-chain rotamer sampling to account for these adjustments. In our case, during the design of new ligands for Pteridine reductase [PTR1, Protein Data Bank (PDB) ID: 3JQA], several side chains of 3JQA adopted new orientations (Fig. 4B) relative to the DX4-bound state (Fig. 4C), forming additional polar contacts while retaining existing π-π interactions with the protein.

To assess whether including the protein binding pocket in the design process improves protein-ligand binding affinity, we computed both AutoDock Vina (Fig. 4D) and MedusaDock scores (Fig. 4E) (*27*). Molecules generated by YuelDesign consistently achieved better scores than those from the other two methods. Redocking (Fig. 4F) the designed molecules with MedusaDock further confirmed that YuelDesign-generated ligands exhibited lower RMSD values, indicating more accurate binding poses.

Last, we evaluated the structural similarity between the designed molecules and the original DX4 ligand using RDKit fingerprints and Tanimoto scores (Fig. 4G). All three methods produced ligands with relatively low similarity to DX4, reflecting the difficulty of sampling similar compounds in a large chemical space. YuelDesign achieved a slightly higher similarity than the other methods.

### The critical role of protein pocket flexibility in molecular design

**Fig. 5.**
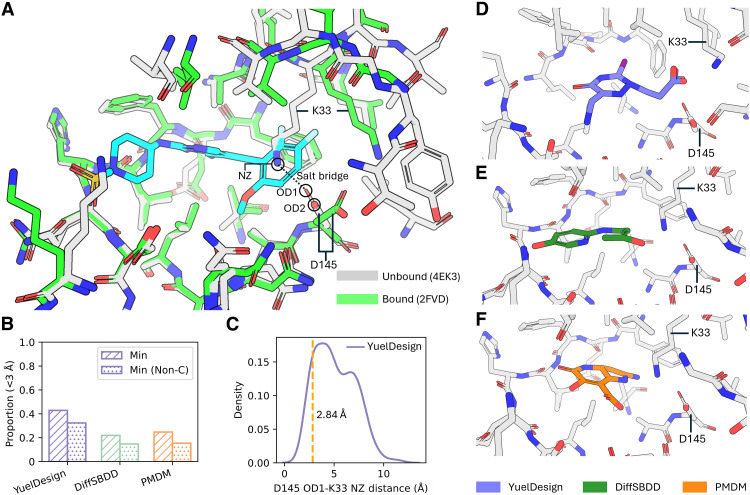
Evaluation of the interactions between CDK2 and generated molecules with different methods. (**A**) Comparison of the pocket structure of the unbound CDK2 (4EK3) and CDK2 bound with diaminopyrimidine inhibitor (PDB ID: 2FVD). A salt bridge is formed between the NZ atom of K33 and OD1 atom of D145 in the unbound CDK2. This salt bridge is broken in the bound state of CDK2. (**B**) Evaluation of the ratio of molecules with the minimum distance to D145 less than 3 Å. “Min” refers to the minimum distance between all atoms, and “Min (Non-C)” refers to the minimum distance between non-carbon atoms. (**C**) Evaluation of the distance between D145 OD1 atom and K33 NZ atom. This distance is fixed in the molecules generated by DiffSBDD and PMDM because these two methods fixed the pocket structure. A 2.84 Å is the distance between the two atoms that form the salt bridge, NZ atom of K33 and OD1 atom of D145. (**D** to **F**) Comparison of the molecules and pocket structures generated by the three methods.

A central objective of YuelDesign is to explicitly model protein pocket flexibility, addressing a key limitation of existing diffusion-based molecular design methods that treat binding pockets as rigid structures. As a use case to demonstrate the impact of incorporating flexibility, we conducted targeted analyses on cyclin-dependent kinase 2 (CDK2), a well-characterized protein with known conformational changes upon ligand binding. CDK2 exhibits a distinct structural transition between unbound (PDB ID: 4EK3) and ligand-bound states: In the unbound form, a salt bridge forms between the NZ atom of lysine-33 (K33) and the OD1 atom of aspartic acid–145 (D145), while this interaction is disrupted in the bound state to accommodate ligand binding (*36*) (Fig. 5A). This conformational switch serves as a sensitive benchmark for evaluating a model’s ability to capture pocket flexibility. Notably, in numerous active CDK2 complexes, including 2FVD (*37*) (CDK2 with a diaminopyrimidine inhibitor), 1DI8 (*38*) (CDK2 bound to a 4-anilinoquinazoline inhibitor), 1KE8 (*39*) (CDK2 complexed with an oxindole-based inhibitor), and 1PYE (*40*) (CDK2 in complex with an aminoimidazo-pyridine inhibitor), the interaction between D145 and the inhibitor becomes noticeably more favorable than the unbound state. D145 frequently acts as a hydrogen bond acceptor with the inhibitor or forms π-H bonds (Fig. 5A) with the ligand’s aromatic rings, highlighting the functional relevance of D145’s conformational adaptability in mediating high-affinity binding.

We first assessed the ability of generated molecules to induce native-like conformational changes in the CDK2 pocket. For each method, we calculated the minimum distance between generated ligands and the D145 residue (Fig. 5B), as close interactions between ligands and D145 are associated with the disruption of the K33-D145 salt bridge and the formation of favorable ligand-D145 contacts observed in native active complexes. YuelDesign-generated molecules showed a noticeably higher proportion of structures with minimum distances to D145 < 3 Å (both for all atoms and noncarbon atoms, which are more likely to form polar or ionic interactions), indicating that these ligands effectively engage with D145 to recapitulate native binding modes. In contrast, molecules from DiffSBDD and PMDM rarely achieved this close proximity.

To directly quantify pocket flexibility, we measured the distance between the K33 NZ atom and D145 OD1 atom in generated complexes (Fig. 5C). DiffSBDD and PMDM do not allow for pocket conformational adjustments, preventing the disruption of the K33-D145 salt bridge, limiting the potential for the favorable D145-ligand interactions observed in native active complexes. In stark contrast, YuelDesign generated complexes with a broad distribution of K33-D145 distances, including values comparable to those in native bound states (e.g., 2.84 Å in 2FVD), demonstrating that the framework enables physiological salt bridge disruption and D145’s conformational adaptation. This flexibility arises from YuelDesign’s joint generation of protein pocket and ligand structures, which allows side-chain rearrangements to coevolve with ligand binding.

The visual inspection of generated complexes further confirmed the importance of pocket flexibility (Fig. 5, D to F). YuelDesign-generated ligands adopted binding poses that form complementary interactions with the rearranged D145 residue. In contrast, ligands from rigid-pocket methods failed to engage D145 because the persistent K33-D145 salt bridge in the fixed pocket creates a steric and electrostatic barrier that hinders productive binding. Collectively, these results highlight that protein pocket flexibility is not merely a structural detail but a critical determinant of successful molecular design.

### Structural evolution during denoising process

**Fig. 6.**
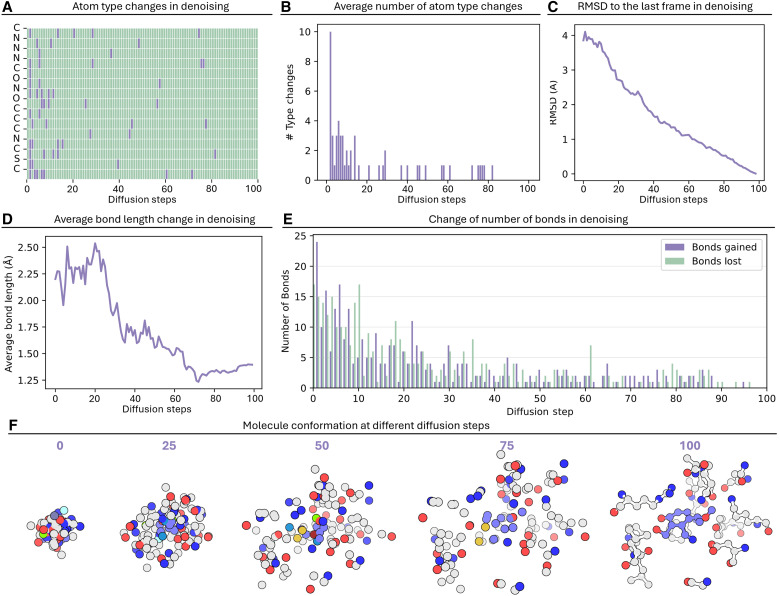
Structural evolution and conformational changes during the denoising process in the generation of PDB 3JQA. (**A**) Atom type changes across diffusion steps for individual atoms, where purple indicates atom type transitions and green represents stable atom types. (**B**) Average number of atom type changes per diffusion step. (**C**) RMSD to the final frame during denoising. (**D**) Evolution of average bond length changes throughout the denoising process. (**E**) Quantification of bond formation (purple) and bond breaking (green) events at each diffusion step. (**F**) Visualization of molecular conformation at representative diffusion steps (0, 25, 50, 75, and 100), showing the progressive refinement from initial random coordinates to the final chemically valid 3D structure. Colored spheres represent different atom types within the evolving.

Last, we conducted a detailed analysis of the structural evolution during the denoising process of the diffusion model, examining both atomic and molecular-level changes (Fig. 6). The analysis is still for pteridine reductase and includes atom-type transitions, structural stability, bond dynamics, and overall conformational changes.

Atom-type evolution analysis demonstrated how atoms changed during the denoising process (Fig. 6A). Individual atoms showed varying degrees of stability, with some maintaining consistent atom types while others underwent transitions before reaching their final states. Notably, most atom types stabilized in the final 20 steps of diffusion, indicating that atom type determination primarily occurs in the later stages of the process. The average number of atom type changes per diffusion step showed a general decreasing trend (Fig. 6B), with initial fluctuations that gradually diminished as the denoising process progressed toward the final molecular structure.

We also calculated the RMSD between atomic coordinates at each denoising step and those in the final structure. Unlike atom-type transitions, we found that RMSD showed a steady and uniform decrease throughout the process. We tracked the formation of bonds by analyzing bond lengths between atoms that form bonds in the final structure. Around step 25, these bond lengths began to consistently decrease, suggesting bond formation and subsequent optimization occurred at this stage. In addition, we recalculated bonds at each diffusion step based on interatomic distances. In the early denoising phase, bond formation and breaking were highly frequent due to the close proximity of atoms sampled from Gaussian noise. Between steps 40 to 90, bond changes showed a stable low fluctuation. In the final 50 steps, bond changes essentially ceased, indicating that the bonding pattern had stabilized.

The overall conformational evolution was visualized at key diffusion steps (0, 25, 50, 75, and 100) (Fig. 6F). The progression from initial random coordinates to the final chemically valid 3D structure was evident. Notably, after step 75, the coordinates remained relatively stable, with subsequent steps primarily focused on fine-tuning atomic positions and adjusting atom types. This observation aligns with our earlier analysis of atom-type transitions and bond dynamics, where we observed similar patterns of stabilization in the later stages of the diffusion process.

## DISCUSSION

The evaluation of YuelDesign shows several key findings about the capabilities and limitations of diffusion-based molecular design. The framework generates chemically valid and structurally stable molecules while maintaining properties relevant for drug discovery applications. The functional group distribution, particularly the balanced representation of common and rare groups, shows that YuelDesign can maintain similarity to known ligands.

A critical challenge in structure-based molecular design is accounting for protein pocket flexibility. Conventional methods, such as DiffSBDD and PMDM, typically treat protein pockets as rigid, which limits their ability to capture induced-fit effects and side-chain rearrangements that occur upon ligand binding. In contrast, YuelDesign simultaneously generates both the protein pocket and small-molecule structures, allowing for dynamic adjustments of side chains. Our RMSD analysis indicates that generated pockets maintain realistic conformations relative to native structures, with a median RMSD of 1.8 Å, which is consistent with the minor adjustments generally observed in protein-ligand binding. In 2008, Brylinski and Skolnick (*41*) analyzed 521 apo-holo protein pairs and showed that 80% had RMSD of ≤1 Å. Studies with Pteridine reductase (3JQA) demonstrate that YuelDesign-generated molecules can form new polar contacts and retain key π-π interactions, leading to improved docking scores and lower RMSD upon redocking compared to molecules designed by other methods. This highlights the advantage of incorporating protein flexibility directly into the generative process.

The E3former architecture in YuelDesign adapts the Evoformer stack to handle equivariant coordinate prediction. By combining sequence, pairwise, and geometric features, E3former enables simultaneous reasoning over protein and ligand atoms while maintaining 3D spatial relationships. The equivariant coordinate head predicts atomic displacements in a rotation- and translation-equivariant manner, producing physically consistent 3D structures. The single-sequence pipeline, together with triangle attention and multiplication modules, propagates structural context across the complex structure without requiring multiple sequence alignments or canonical residue ordering.

YuelDesign uses two diffusion models to generate molecules: EDM for continuous coordinates and D3PM for discrete atom types. EDM gradually refines atomic coordinates through a denoising process that balances noise injection and signal preservation, while D3PM models the evolution of discrete atom types via a Markov process over categorical states. The joint training of EDM and D3PM on shared E3former features allows the model to learn correlations between geometry and atom identity, generating molecules in which atom types are consistent with local structural environments. This combination helps maintain chemical validity, connectivity, and compatibility between the ligand’s structure and its atom types during generation.

While YuelDesign shows clear advantages, several limitations remain. First, the similarity to native ligands, although improved relative to other methods, is still modest, indicating that the vast chemical space remains challenging to explore fully. Second, small rings and other strained motifs are overrepresented due to the lack of explicit chemical enforcement during initial sampling. Third, the performance of the diffusion model tends to decrease for larger molecules. One factor is the increased dimensionality of the molecular representation. In our current framework, each atom is represented by both its 3D coordinates and feature vector, and the diffusion process operates on all atoms simultaneously. As the number of atoms increases, the total dimensionality of the input grows, which can make the denoising task more challenging. High-dimensional data can exacerbate learning difficulties, including slower convergence and accumulation of small errors during the iterative denoising steps, leading to lower validity or connectivity for larger molecules. A potential strategy to mitigate this issue is to adopt a latent diffusion approach. By first mapping molecules into a lower-dimensional latent space, the model can operate in a more compact and structured representation, reducing the effective dimensionality of the diffusion process. This could make the denoising more stable and scalable, particularly for larger molecules, while preserving the essential structural and chemical information. Future work could explore this latent diffusion framework to improve generation quality for high-dimensional, large-molecule systems.

Last, the analysis of the denoising process shows how diffusion models refine molecular structures. The patterns in atom type transitions, bond dynamics, and conformational changes reveal a systematic approach to structure refinement. The stabilization of coordinates after step 75, combined with the continued fine-tuning of atomic positions and atom types, suggests that the model uses a hierarchical approach to structure generation. This understanding could guide future optimizations, such as decoupling atom-type determination from 3D coordinate refinement, potentially improving denoising efficiency.

## MATERIALS AND METHODS

### Overview of the molecular generation framework

Please refer to Supplementary Methods for a detailed description of the YuelDesign architecture. In this and the following sections, we will provide a concise overview of its main components.

YuelDesign generates molecules conditioned on a protein pocket structure by jointly modeling atomic coordinates and atom types. The core of the model is the E3former, an Evoformer-based architecture adapted to process sequence, pairwise, and coordinates while preserving 3D rotational and translational equivariance. The equivariant coordinate head predicts atomic displacements in a manner that maintains physically consistent structures.

The generation process relies on two diffusion models: EDM (*31*) for continuous atomic coordinates and D3PM for discrete ligand atom types. During training, EDM learns to denoise molecular coordinates corrupted with Gaussian noise, while D3PM predicts the original atom types from discretely noised categorical states. Both models share the same E3former features and time steps, allowing the network to learn correlations between geometry and chemical identity. Sampling starts from random noise in both continuous and discrete spaces and proceeds through iterative denoising to produce chemically valid molecules with proper connectivity.

### Data preparation

Binding MOAD (Mother of All Databases) (*42*) is a carefully curated repository of high-quality protein-ligand crystal structures drawn from the PDB (*43*), designed to support structure-based drug discovery and polypharmacology investigations. The database has about 41,409 structural entries, with quantitative affinity measurements available for 15,223 (37%) of them, making it one of the most extensive resources for probing molecular recognition in biological systems. We prepared the MOAD dataset using a multistep processing pipeline. For small molecules, we extract atomic features using RDKit, where each atom is represented by its 3D coordinates and a one-hot encoded feature vector. The atomic feature only takes the atom type (tables S1 and S2). We split the dataset to training and testing sets with a ratio of 8:2. To minimize leakage from homologous or highly similar protein pockets, we implemented a multilevel similarity filtering procedure. We computed pairwise protein sequence similarity using BLASTp (*44*) and assessed structural similarity of binding pockets with TM-align (*45*) and local RMSD of pocket residues. We assigned complexes with sequence identity greater than 30% or pocket RMSD below 2 Å to the same split. For ligands, we calculated the Tanimoto similarity using RDKit fingerprints and assigned highly similar ligands (Tanimoto of >0.85) to the same split. This approach prevents highly similar proteins or ligands from appearing across training and test sets, effectively reducing the risk of data leakage.

The 3D structural information is preserved through the spatial coordinates of both protein and molecular atoms. We define the binding site as all protein residues that have at least one atom within 6 Å of any atom of the ligand. This distance-based criterion identifies residues that are spatially close enough to potentially interact with the ligand, either through direct contacts such as hydrogen bonds, hydrophobic interactions, or π-π stacking, or indirectly by influencing the shape and chemical environment of the binding pocket. The molecular and protein coordinates are concatenated to form a single coordinate matrix while maintaining the distinction between pocket and molecular atoms through binary masks.

For each protein-ligand complex, we construct detailed sequence and pair features to encode both chemical and geometric information. Each atom in the protein and ligand is treated as a token in the sequence, with a categorical indicator distinguishing protein backbone atoms, side chain, and ligand atoms. For the sequence features, we generate a one-hot encoding of allowed atom types, including virtual ring center atoms for aromatic residues and a generic placeholder type for unknown atoms. In addition to chemical information, we encode spatial and relational context through pairwise features. For each pair of atoms, we include a binary indicator of whether the two atoms belong to the same residue, providing local structural context. We also compute the Euclidean distance between the atoms, which supplies translationally invariant geometric information. All the features are summarized in table S3.

### E3former architecture

The core of YuelDesign is the E3former, an adaptation of Evoformer stack designed for molecular complexes. E3former operates on both sequence features, representing atoms, and pair features, representing relationships between atom pairs, to iteratively refine atomic coordinates and embeddings. Unlike the original Evoformer, E3former omits Multiple Sequence Alignment (MSA) inputs and positional encodings, as protein pockets and ligands lack canonical ordering, and instead relies on single-sequence attention.

Within each E3former block, sequence attention, sequence transition, and sequence outer product modules propagate information among atoms. Pair features are updated through triangle attention and triangle multiplication modules, which model geometric relationships and enable long-range interactions. Pair transition layers provide additional nonlinear transformations on pairwise embeddings.

E3former also includes an equivariant coordinate head that converts the final pair embeddings into coordinate displacements. This module aggregates weighted direction vectors between atoms, ensuring that the predicted updates are equivariant to rotations and translations. As a result, E3former can directly refine atomic positions in 3D space while simultaneously updating sequence features for downstream diffusion models.

### Diffusion model

YuelDesign employs a dual diffusion framework to generate molecular structures, simultaneously modeling continuous 3D coordinates and discrete atom types. Continuous features represent atomic positions in Cartesian space, while discrete features encode atom types using one-hot vectors. The continuous diffusion process follows the EDM framework. During forward diffusion, Gaussian noise is gradually added to atomic coordinates according to a predefined signal-to-noise ratio schedule, producing a sequence of increasingly noisy structures. The model is trained to predict the added noise, enabling the reverse process to iteratively denoise and recover the original coordinates. The forward and reverse processes are formally expressed as

$$x_{t}=\alpha_{t}x_{0}+\sigma_{t}\epsilon ,\;\epsilon ∼𝒩\;\left(0,I\right)$$

where $x_{t}$ is the atomic coordinates at the time step *t*, $\alpha_{t}$ and $\sigma_{t}$ are noise scaling factors derived from the schedule, and ϵ is Gaussian noise. The model learns a noise prediction network and training minimizes the masked mean squared error between predicted and true noise, ensuring accurate score estimation for denoising.

For discrete atom types, YuelDesign uses D3PM. The forward process progressively corrupts atom-type labels through stochastic transitions governed by a transition probability $\beta_{t}$, while the reverse process learns to recover the original categorical distribution of atom types. This approach allows the model to handle the mixed continuous-discrete nature of molecular structures, ensuring chemical plausibility in generated ligands.

Both EDM and D3PM are conditioned on features derived from the receptor-ligand complex, which are processed by the E3former network. E3former updates node features and coordinates while maintaining rotational and translational equivariance, leveraging attention and triangle-based operations to propagate geometric and relational information. During generation, the model starts from random noise for both coordinates and atom types and iteratively refines the structure through the learned reverse diffusion processes. The final output consists of a complete 3D pocket structure along with the generation trajectory if needed.

### Chemical properties evaluation

We evaluated the generated molecules using multiple metrics, including QED, Lipinski’s RO5, SAS, and validity checks. Detailed descriptions of these metrics, including the specific implementation of the SAS score calculation, are provided in Supplementary Methods.

### Functional group analysis

The functional group analysis employs a comprehensive set of SMARTS (*46*) patterns (table S4) to identify various chemical functional groups in the generated molecules. The analysis covers a wide range of functional groups including carboxylic acids, esters, amides, ketones, aldehydes, various types of amines, alcohols, phenols, ethers, epoxides, thiols, thioethers, sulfonamides, halogens, and various aromatic and special ring structures. Each functional group is defined using SMARTS, which are powerful molecular pattern-matching expressions. For example, carboxylic acids are identified using the pattern “C (═O)[OH],” while primary/secondary amines use “[NX3;H2,H1;!$ (NC═O)].” The frequency calculation is performed by counting the occurrences of each functional group across all molecules in the dataset.

### Structural analysis

The structural analysis of generated molecules includes three key aspects: bond length distributions, atom type transitions, and conformational changes. Bond length analysis focuses on the evolution of interatomic distances throughout the diffusion process. Using a 1.9-Å cutoff for covalent bond identification, the analysis tracks individual bond distances across all diffusion steps. Atom-type transitions are analyzed through a systematic tracking of changes in atomic identities during the diffusion process. The analysis uses a binary matrix representation, where each element indicates whether an atom’s type changed at a particular diffusion step. The analysis also quantifies the frequency of type changes per step and identifies atoms that undergo the most transitions. Conformational analysis uses multiple metrics to assess structural evolution. The RMSD between intermediate and final structures quantifies the magnitude of conformational changes. In addition, the analysis tracks bond dynamics by monitoring both the total number of bonds and the changes in bond counts between consecutive steps.

### Protein-ligand interactions analysis

Docking energy is evaluated using MedusaDock scores, which quantify the strength of protein-ligand interactions. The analysis compares docking scores between generated molecules and native ligands across different molecular size ranges (e.g., 11 to 15 and 16 to 20 atoms). The score distributions are visualized using kernel density estimation plots, where lower (more negative) scores indicate stronger binding affinity. This analysis helps understand how the model balances molecular size with binding strength. Molecular similarity analysis was performed using RDKit’s default Daylight-like fingerprints. Each molecule is represented as a binary vector encoding the presence of topological substructures, and pairwise similarity between molecules is quantified using the Tanimoto coefficient.

## Supplementary Materials

### The PDF file includes:

Supplementary Methods Tables S1 to S4 Legend for movie S1 References
