# Supplementary material for: A diffusion-based framework for designing molecules in flexible protein pockets
Source: Sci Adv. 2026 Apr 8;12(15):eaeb7045. doi: 10.1126/sciadv.aeb7045 (PMC13060587; doi:10.1126/sciadv.aeb7045)
Supplement: Supplementary file 1 — Supplementary Methods Tables S1 to S4 Legend for movie S1 References [file sciadv.aeb7045_sm.pdf]

Science Advances
AAAS

Supplementary Materials for

A diffusion-based framework for designing molecules in flexible protein pockets

Jian Wang *et al.*

Corresponding author: Nikolay V. Dokholyan, dokh@virginia.edu

Sci. Adv. **12**, eaeb7045 (2026)
DOI: 10.1126/sciadv.aeb7045

The PDF file includes:

Supplementary Methods
Tables S1 to S4
Legend for movie S1
References

Other Supplementary Material for this manuscript includes the following:

Movie S1

Supplementary Methods

YuelDesign couples two diffusion models, EDM for continuous coordinates generation and D3PM for discrete atom types generation, with E3former, an Evoformer-based equivariant coordinates predictor. The pipeline consumes receptor pockets and ligands, encodes them into node and pair tensors, and refines both coordinates and discrete atom identities through an E3former block. We will first enumerate the feature pipeline and atom vocabularies, then describe the E3former architecture, the equivariant coordinate head, and the diffusion models (EDM for coordinates, D3PM for ligand atoms) that drive training and sampling.

Feature Construction

Each training example is sourced from the MOAD pocket database, pairing a receptor pocket PDB with its ligand MOL block. In the receptor preprocessing stage, we retain all non-hydrogen atoms, guaranteed `CA` positions, and virtual ring-center atoms for aromatic residues. This reduced coordinate set defines the receptor nodes, to which ligand atoms are appended, yielding a total of $N = N_{\text{receptor}} + N_{\text{ligand}}$ mixed nodes.

The node encoder constructs features $h \in \mathbb{R}^{N\times 88}$ by concatenating a 3-way molecule-type indicator (`[backbone, side_chain_or_ring, ligand]`) with an 85-way one-hot encoding over `ALLOWED_ATOM_TYPES` (comprising `'X'` plus every non-carbon protein atom and ring center), matching `in_node_features` $= 88$. Pair features $z \in \mathbb{R}^{N\times N\times 1}$ record residue membership as a single scalar per node pair: 1 if both indices belong to the same protein residue (with ligand atoms assigned index -1), and 0 otherwise. During the forward pass, `E3former` concatenates Euclidean distances, so the embedding layer ultimately receives two channels: `[is_same_residue, distance]`. Receptor and ligand coordinates $x \in \mathbb{R}^{N\times 3}$ are concatenated prior to modeling to seed the E3 head, with all positions maintained in Ångströms.

Supervision and masking are handled through several tensors: `receptor_mask` distinguishes receptor nodes (1) from ligand nodes (0); `anchor_mask` identifies receptor `CA` atoms (1) used for recentering structures; and `seq_mask`/`pair_mask` allow padding-aware batching (defaulting to all-ones for unpadded samples). Ligand atom classes are stored in `ligand_atoms` as indices over `LIGAND_ATOM_TYPES` (totaling `num_ligand_atom_types` $= 20$), while `receptor_interaction` highlights receptor atoms within 5 Å of any ligand heavy atom for downstream filtering.

Before entering E3former, the model assembles the sequence tensor by concatenating additional conditioning to h:

$$\text{seq} = [h,\ \text{onehot}(x_t^{\text{atoms}}),\ \text{anchor\_mask},\ t],$$

yielding `seq_input_dim` $= \text{in\_node\_features} + \text{num\_ligand\_atom\_types} + 2 = 88 + 20 + 2 = 110$. The ligand one-hot vector is zeroed on receptor nodes to preserve mixed graph semantics. Unless stated otherwise, experiments use `n_blocks` $= 8$, `hidden_nf` $= 64$, `no_heads_seq` $= 4$, `no_heads_pair` $= 2$, `transition_n` $= 4$, `blocks_per_ckpt` $= 4$, and `chunk_size` $= 4$. Pair tensors feed into representations of width $c_m = c_z = \text{hidden\_nf}$, and the single-head output

dimension is c_s = num_ligand_atom_types. The model was trained using the AdamW optimizer with a learning rate of 0.0002.

Overall Architectural

E3former extends AlphaFold's Evoformer stack, inheriting all pairwise operations, triangle modules, and transition layers unchanged while adapting inputs and heads for molecular systems. Batches contain single-chain features $seq \in \mathbb{R}^{B\times N\times C_{\text{seq}}}$, Cartesian coordinates $x \in \mathbb{R}^{B\times N\times 3}$, pair descriptors $z \in \mathbb{R}^{B\times N\times N\times C_{\text{pair}}}$, sequence masks m_{seq}, and pair masks m_{pair}. Since molecule atoms and pocket residues have no canonical ordering, positional encodings and MSA features are omitted.

The original MSA stack is replaced with a single-sequence pipeline (SequenceAttention → SequenceTransition → SequenceOuterProduct) that conditions on the pair tensor, marking the primary architectural deviation inside the trunk. In the embedding stage, seq is linearly projected to c_m, while pair features concatenate scalar descriptors with Euclidean distances prior to projection into c_z. The `EvoformerStack` executes `no_blocks` residual blocks using the single-sequence module plus the inherited pair/triangle operations, emitting updated m, z, and an auxiliary single embedding s. Finally, a dedicated equivariant head consumes the final pair tensor z_{out} to generate coordinate displacements Δx, producing refined coordinates x'. This head is absent in the original Evoformer.

Input Geometry Encoding

For each structure we compute dense geometric descriptors:

$$\Delta x_{ij} = x_i - x_j, \qquad d_{ij} = \| \Delta x_{ij} \|_2,$$

$$\hat{u}_{ij} = \frac{\Delta x_{ij}}{d_{ij} + \epsilon},$$

with $\epsilon = 10^{-8}$ for stability. Distances are appended as an extra scalar channel before the pair projection, ensuring that translationally invariant information is available to the Evoformer.

Evoformer Block Composition

SequenceAttention

Without MSAs, contextualization must arise from the single chain itself. SequenceAttention mirrors the AlphaFold single-chain ablation, letting each residue attend to all others while injecting relational bias from z. The sequence and pair tensors m and z are layer-normalized to stabilize statistics without positional encodings. Projections $q = W_q m$, $k = W_k m$, and $v = W_v m$ are reshaped into `no_heads_seq` heads of width `c_hidden_seq_att`. The pair bias `Linear_z`(z) yields per-head logits b_{ijh}, supplying the geometric cues that positional encodings would otherwise provide. Attention logits are computed as $\alpha_{ijh} = (q_{ih} \cdot k_{jh})/\sqrt{c_{\text{hidden}}} + b_{ijh}$, with masks contributing $-\infty$ where residues or atoms are invalid. Output gating $g = \sigma(W_g m)$ modulates the

attended values before the residual projection W_o. Combined with the downstream modules it forms the "Sequence module," the first major departure from Evoformer.

SequenceTransition

SequenceTransition implements the equivalent of Algorithm 9, supplying feed-forward capacity for per-residue channel mixing. Structurally, it consists of LayerNorm followed by an expansion `Linear`$(c_m, n \cdot c_m)$, a ReLU activation, and a compression `Linear`$(n \cdot c_m, c_m)$, with final masking applied by m_{seq}. The module uses He initialization in the expansion layer and zero-initialized final weights (`init="final"`) to ensure controlled residual dynamics.

SequenceOuterProduct

SequenceOuterProduct replenishes the pair tensor using single-sequence information, compensating for the absence of MSAs. It takes layer-normalized sequence activations and masks as input. Two independent linear maps produce a_i and b_i in $\mathbb{R}^{c_{\text{hidden\_opm}}}$. The outer product $O_{ij} = a_i \otimes b_j$ is computed and reshaped to $\mathbb{R}^{c^2_{\text{hidden\_opm}}}$ before being projected back to c_z. Outputs are divided by the valid pair count to maintain scale when atom counts vary.

Triangle Attention

Both `TriangleAttentionStartingNode` and `TriangleAttentionEndingNode` are identical to the AlphaFold implementations (Algorithms 13–14). They apply multi-head attention over triangular walks in the pair graph to disseminate context. Readers can refer to the AlphaFold supplement for the derivations; E3former inherits the modules as-is aside from optional masking hooks.

Triangle Multiplication

`TriangleMultiplicationOutgoing` and `TriangleMultiplicationIncoming` likewise replicate Algorithms 11–12 from AlphaFold, contracting along shared triangle indices with gated projections. Their full descriptions, including tensor diagrams, follow exactly the supplemental material of the AlphaFold paper; E3former only exposes optional masks to remain faithful to pocket-focused inputs.

PairTransition

PairTransition acts as Algorithm 15, providing per-edge nonlinear mixing that complements the triangle modules. The pipeline consists of LayerNorm followed by `Linear`$(c_z, n \cdot c_z)$, a ReLU activation, and `Linear`$(n \cdot c_z, c_z)$ with optional mask multiplication. Unlike DeepMind's original release, this implementation optionally multiplies by m_{pair} to enforce sparsity when required.

Overall, these modules follow the AlphaFold supplement algorithms but are streamlined for the single-sequence scenario used by E3former.

E3 Coordinate Module

The second major deviation from Evoformer is the equivariant coordinate head appended after the trunk. Given the final pair activations z_{out}, it predicts coordinate displacements instead of passing z to a structure module:

$$w_{ij} = \text{MLP}(z_{ij}^{\text{out}}) \in \mathbb{R}, \qquad \delta x_i = \sum_j w_{ij}\ \hat{u}_{ij}, \qquad x_{i'} = x_i + \delta x_i.$$

Key properties of this module include the use of direction fields, scalar weights, and aggregation. `coord2diff` computes normalized direction vectors $\hat{u}_{ij}$ once per batch, ensuring translation invariance and rotation equivariance. The `coord_mlp` (a three-layer MLP with SiLU activations) maps each pair channel to a scalar w_{ij}; no positional indices are needed because geometry is conveyed through z_{out} and distances. Summing the weighted direction vectors over j yields per-residue displacements δx_i, and residual addition produces the updated coordinates x'. Sequence or pair masks can zero out invalid contributions (e.g., truncated pocket residues or dummy atoms) before the summation. This head allows E3former to emit refined atomic positions directly, a capability not present in bare Evoformer.

Diffusion Models

YuelDesign employs dual diffusion processes for joint coordinate and atom-type refinement. Diffusion models provide a principled framework for learning data distributions through a gradual noising and denoising process, offering stable training dynamics and high sample quality. Unlike autoregressive models that impose strict ordering constraints, diffusion models allow flexible conditional generation and naturally handle the mixed continuous-discrete nature of molecular structures. Continuous coordinate updates follow the Elucidated Diffusion Model (EDM), which unifies variance-preserving and variance-exploding formulations through a parameterization that enables optimal training and sampling. Discrete ligand atom categories evolve via a Discrete Denoising Diffusion Probabilistic Model (D3PM), which extends diffusion principles to categorical data through Markov chain transitions. Both processes share the same timestep t, enabling the network to learn correlated coordinate and atom-type dynamics through joint training.

EDM Continuous Coordinates

The coordinate diffusion process follows the Elucidated Diffusion Model parameterization, which provides a unified framework for continuous diffusion by expressing the forward process through signal-to-noise ratio (SNR) scales. The EDM parameterization elegantly bridges variance-preserving (VP) and variance-exploding (VE) diffusion formulations, allowing adaptive noise schedules that optimize both training stability and sample quality. For $T =$ diffusion_steps, we employ a polynomial schedule

$$\bar{\alpha}_t = \left(1 - \left(\frac{t}{T+1}\right)^2\right)^2$$

which provides smooth interpolation from clean data ($t = 0$) to pure noise ($t = T$). This polynomial form ensures that the noise level changes gradually during both early and late stages of diffusion, avoiding sharp transitions that can destabilize training. The schedule is discretized into multiplicative steps and clipped by the precision constant ($\texttt{diffusion\_noise\_precision} = 10^{-5}$) to prevent numerical degeneracy when $\bar{\alpha}_t \approx 0$ or $\bar{\alpha}_t \approx 1$.

The EDM framework operates in log-SNR space, where each timestep stores $\gamma_t = -\log \alpha_t^2/\sigma_t^2$. This representation provides a compact and numerically stable encoding of the noise schedule. The signal and noise scales are recovered through closed-form expressions

$$\alpha_t = \sqrt{\sigma(-\gamma_t)}, \qquad \sigma_t = \sqrt{\sigma(\gamma_t)},$$

where σ denotes the logistic sigmoid function. The log-SNR γ_t directly controls the information content: large negative values keep samples close to clean coordinates with minimal noise, while positive values inject substantial Gaussian noise. This parameterization automatically ensures that $\alpha_t^2 + \sigma_t^2 = 1$ through the sigmoid relation, maintaining a natural balance between signal preservation and noise injection.

During forward diffusion, given centered target coordinates x_0, noisy samples are drawn as

$$x_t = \alpha_t x_0 + \sigma_t \epsilon, \qquad \epsilon \sim \mathcal{N}(0, \mathbf{I}),$$

where ϵ represents independent Gaussian noise. The forward process creates a sequence of increasingly noisy configurations, forming a Markov chain that gradually corrupts structural information. Optionally, anchor atoms (backbone CA) remain noise free during forward diffusion, preserving receptor geometry as structural constraints. This masking strategy leverages the known receptor structure to guide ligand generation while still allowing the model to learn from complete noisy configurations.

For training, the model learns to reverse this forward diffusion process by predicting the score-equivalent residual $\hat{\epsilon}(x_t, t)$. The training objective employs a masked ℓ_2 loss $\| \hat{\epsilon} - \epsilon \|^2$, which corresponds to score matching in the EDM framework. Score matching provides a principled approach to learning distributions by modeling the gradient of the log-probability density (the score function), avoiding the need for explicit likelihood computation. The residual prediction $\hat{\epsilon}$ directly estimates the noise added during forward diffusion, enabling exact reconstruction via $x_0 = (x_t - \sigma_t \hat{\epsilon})/\alpha_t$. Timestep sampling weights follow $p(t) \propto t^{\text{t\_weight\_power}}$ (default 0, corresponding to uniform sampling), which can be adjusted to bias loss contributions toward later timesteps when finer denoising details are more critical.

D3PM Discrete Atom Types

Ligand atom categories ($K = \text{num\_ligand\_atom\_types} = 20$) evolve via a Discrete Denoising Diffusion Probabilistic Model, which extends diffusion principles to discrete categorical data through Markov chain transitions over a finite state space. Unlike continuous diffusion where noise is additive Gaussian, discrete diffusion operates through stochastic transitions between categorical states. D3PM employs a uniform transition matrix that provides symmetric transitions between all atom types, ensuring unbiased exploration of the categorical space.

The diffusion schedule follows a cosine decay over the discrete timesteps. The transition probability β_t is derived from a cosine schedule as

$$\beta_t = \min\left(1 - \frac{\bar{\alpha}_t}{\bar{\alpha}_{t-1}}, 0.999\right)$$

where $\bar{\alpha}_t = \cos^2(\frac{(t+0.008)\pi}{2(1.008)})$ provides smooth decay from 1 to 0 over $t \in [0, T]$. The small offset 0.008 prevents numerical issues at the boundaries, and the cosine form ensures gradual transitions that match the continuous coordinate process. This defines the one-step transition kernel

$$q(x_t = j \mid x_{t-1} = i) = \begin{cases} 1 - \dfrac{K-1}{K}\beta_t & i = j, \\ \dfrac{1}{K}\beta_t & i \neq j. \end{cases}$$

This formulation ensures detailed balance and symmetry: each atom type has probability $\frac{K-1}{K}\beta_t$ of transitioning to a different type, uniformly distributed among the remaining $K - 1$ options, and probability $1 - \frac{K-1}{K}\beta_t$ of remaining unchanged. As β_t increases from 0 to 1, the process gradually erases categorical information, with the limiting distribution $q(x_T \mid x_0)$ approaching the uniform prior over all K atom types. The slightly higher self-transition probability maintains stability during early diffusion steps while allowing efficient exploration of the categorical space.

The multi-step forward distribution $q(x_t \mid x_0)$ is obtained through cumulative products of the transition matrices, computed efficiently using matrix exponentiation. This closed-form expression enables exact computation of the forward process at any timestep without sequential sampling. During forward diffusion, sampling uses Gumbel perturbations to draw discrete indices, ensuring unbiased categorical sampling without introducing continuous relaxations that could distort the discrete nature of the problem.

For denoising, the model learns to predict $p_\theta(x_0 \mid x_t)$ (realized as s_{out}), which estimates the original atom type distribution given the corrupted state. The optimal backward kernel for discrete diffusion satisfies

$$q(x_{t-1} \mid x_t, x_0) \propto q(x_t \mid x_{t-1}) q(x_{t-1} \mid x_0),$$

which follows from Bayes' rule and the Markov property of the forward process. This posterior distribution can be evaluated in closed form using the precomputed transition matrices and forward distributions, enabling exact likelihood computation for training. The model approximates this optimal backward process by predicting the clean data distribution $p_\theta(x_0 \mid x_t)$ and using it to compute the posterior via the same relation, effectively learning to reverse the forward Markov chain.

Training minimizes cross-entropy between the predicted logits and the clean ligand labels, masking out receptor atoms so that only ligand positions contribute to the loss. This masking ensures that the model focuses on learning ligand atom type distributions while receptor atoms

remain fixed. The discrete nature of atom types naturally requires categorical loss functions, making cross-entropy the appropriate choice that aligns with maximum likelihood estimation under the multinomial distribution.

The joint training of coordinate and atom-type diffusion processes is facilitated by conditioning both on the same E3former features and timestep t. The shared representation allows the model to learn correlations between geometric configurations and chemical identities, reflecting the physical principle that molecular geometry and atom types are intrinsically coupled. This joint inference enables the model to generate chemically plausible ligands where atom types are consistent with their local geometric environments.

Training Losses

Let M_{coord} denote the binary mask selecting non-anchor atoms (`sample_mask`). For each timestep the model predicts noise residuals $\hat{\epsilon}$ from x_t. The masked mean-squared error

$$\mathcal{L}_{\text{coords}} = \frac{1}{\sum M_{\text{coord}}} \sum_i M_{\text{coord},i} \parallel \hat{\epsilon}_i - \epsilon_i \parallel_2^2$$

matches the EDM score-matching objective and encourages accurate force-like updates only where supervision exists. For the atom-type loss, let M_{lig} mark ligand nodes (the complement of the receptor mask). Given logits s_{out} and clean labels y, the loss is the masked cross-entropy

$$\mathcal{L}_{\text{atoms}} = -\frac{1}{\sum M_{\text{lig}}} \sum_i M_{\text{lig},i} \log p_\theta(y_i \mid x_t, t),$$

where p_θ is obtained by softmaxing s_{out}. Only ligand positions contribute, so receptor tokens remain unconstrained. Training minimizes $\mathcal{L} = \mathcal{L}_{\text{coords}} + \mathcal{L}_{\text{atoms}}$. Both terms use the same sampled timestep t, ensuring consistent supervision for the coupled continuous and discrete processes.

Bond Determination and Format Conversion

The YuelDesign framework operates by directly generating 3D coordinates and atom types for the ligand molecules, which are initially saved in PDB format. In this representation, bonds are not explicitly defined but are inferred based on the spatial arrangement of atoms. To facilitate downstream analysis and compatibility with various molecular modeling tools, we convert the generated PDB files into MOL2 format using PyMOL (47). PyMOL infers chemical connectivity by calculating interatomic distances and establishing bonds between atoms that fall within standard covalent bonding thresholds (typically around 1.9 Å). While our pipeline officially supports output in PDB format, the generated structures can also be processed and converted using other standard cheminformatics tools such as OpenBabel (48), which similarly rely on distance-based heuristics to reconstruct bond topology from atomic coordinates.

Chemical Properties Evaluation

All evaluations were performed on the Binding MOAD test set. For each protein in the test set, we generated one molecule per method - YuelDesign, DiffSBDD, and PMDM - for each target size ranging from 15 to 35 heavy atoms. The compound size is defined as the number of heavy atoms

in the molecule. These details and the criteria for generating molecules have been added to the Methods section for clarity.

The validity metric serves as a foundational assessment to verify a molecule's chemical plausibility, implemented using the validate function from the MolVS (34) (Molecular Validation and Standardization) toolkit. The MolVS Validator, through its built-in Validations, performs several checks to identify potentially problematic features in a molecule, and logs these findings without making any structural modifications. Specifically, for Charge State, the NeutralValidation records an informational log message if the molecule is not an overall neutral system, meaning its net charge is not zero. Regarding Isotopes, the IsotopeValidation checks and logs if the molecule contains any isotopes, which are atoms with non-natural abundance. For Fragments, the FragmentValidation checks for the presence of certain predefined "fragments," which are typically salts or solvents, recording their existence based on default FragmentPatterns. Furthermore, the SmartsValidation serves as an abstract base class, allowing for more specific checks, such as the DichloroethaneValidation example provided, which logs the presence of 1,2-dichloroethane, demonstrating how users can implement custom validations based on specific SMARTS patterns for any substructure. A molecule is classified as valid if the validate function returns no issues (i.e., an empty list of validation errors). The validity assessment yields a binary outcome (valid/invalid), with "valid" assigned exclusively to molecules that pass all MolVS validation checks and exhibit full structural connectivity.

The connectivity metric evaluates whether all atoms in the molecule are connected through a path of bonds. This is implemented by constructing a graph representation of the molecule where atoms are nodes and bonds are edges. The metric uses NetworkX's *is_connected* function to check if there exists a path between any pair of atoms in the graph. The connectivity check is also binary, returning True if the molecule is fully connected and False if there are disconnected components.

The large ring rate metric identifies molecules containing rings with more than 6 atoms. This is calculated using RDKit's *GetSymmSSSR* (Smallest Set of Smallest Rings) function, which finds all rings in the molecule. The metric returns True if any ring has more than 6 atoms, and False otherwise. This is important because large rings are often undesirable in drug-like molecules due to their potential impact on molecular properties and synthetic accessibility.

The QED metric is a continuous measure that evaluates how drug-like a molecule is. It is calculated using RDKit's *QED.default* function, which considers multiple molecular properties including molecular weight, logP, number of hydrogen bond donors and acceptors, and other structural features. The QED score ranges from 0 to 1, where higher values indicate more drug-like molecules. The formula for QED is:

$$QED = \prod(w_i * p_i)$$

where w_i are weights for different properties and p_i are the property values normalized to [0,1].

The SAS (Synthetic Accessibility Score) (33) metric estimates how difficult it would be to synthesize a molecule. It is calculated using a custom implementation that considers factors such

as ring complexity, stereochemistry, and fragment contributions. The SAS score typically ranges from 1 to 10, where lower values indicate easier synthesis. The score is calculated as:

$$SAS = 1 + \sum(w_i * f_i)$$

where w_i are weights for different complexity factors and f_i are the individual factor scores. Details of the SAS individual factor scores can be found in the next section.

The Lipinski metric evaluates whether a molecule follows the RO5 criteria for drug-likeness. A molecule passes the Lipinski check if it satisfies all of the following conditions: molecular weight ≤ 500, logP ≤ 5, number of hydrogen bond donors ≤ 5, and number of hydrogen bond acceptors ≤ 10. The metric is binary, returning True if all criteria are met and False otherwise. This is implemented using RDKit's *Descriptors* and *Lipinski* functions to calculate the individual properties.

SAS Score Calculation

The SAS score is computed as the sum of three components: fragment score, features score, and fingerprint density correction. The fragment score (score_1) is based on Morgan circular fingerprints and a pre-trained fragment scoring database. The calculation proceeds in three steps. First, a Morgan fingerprint is generated. Next, all non-zero fingerprint elements (fragments) are extracted. Finally, the weighted average fragment score is calculated as:

$$\textit{score}_1 = \frac{\sum_i w_i \times \textit{fragment\_score}_i}{\sum_i w_i}$$

where w_i is the frequency of fragment i in the molecule, fragment_score_i is the pre-trained score for fragment i, and unknown fragments are assigned a default score of -4.

The features score (score_2) accounts for molecular complexity through various penalty terms:

$$\text{score}_2 = 0 - \text{sizePenalty} - \text{stereoPenalty} - \text{spiroPenalty} - \text{bridgePenalty} - \text{macrocyclePenalty}$$

The size penalty is computed as:

$$\textit{SizePenalty} = n_{\text{atoms}}^{1.005} - n_{\text{atoms}}$$

The stereo penalty follows:

$$\textit{StereoPenalty} = \log_{10}(n_{\text{chiral}} + 1)$$

where n_{chiral} is the number of chiral centers. Similarly, the spiro penalty is:

$$\textit{SpiroPenalty} = \log_{10}(n_{\text{spiro}} + 1)$$

where n_{spiro} is the number of spiro atoms. The bridge penalty is:

$$\textit{BridgePenalty} = \log_{10}(n_{\text{bridgeheads}} + 1)$$

where $n_{\text{bridgeheads}}$ is the number of bridgehead atoms. For the macrocycle penalty:

$$\text{MacrocyclePenalty} = \begin{cases} \log_{10}(2) & \text{if } n_{\text{macrocycles}} > 0 \\ 0 & \text{otherwise} \end{cases}$$

where $n_{\text{macrocycles}}$ is the number of rings with size > 8 atoms. This differs from the original paper, which used $\log_{10}(n_{\text{macrocycles}} + 1)$. The modified form generates better results when 2 or more macrocycles are present.

The fingerprint density correction (score$_3$) accounts for molecular symmetry (added in version 1.1 to make highly symmetrical molecules easier to synthesize). The correction is calculated as:

$$\text{score}_3 = \begin{cases} 0.5 \times \log\left(\frac{n_{\text{atoms}}}{|\text{fps}|}\right) & \text{if } n_{\text{atoms}} > |\text{fps}| \\ 0 & \text{otherwise} \end{cases}$$

where |fps| is the number of unique fingerprint bits (fragment types).

Table S1. Protein Atom Vocabulary

Category	Supported atom names
Carbon backbone/side-chain	CA, CB, CG, CD, CE, CZ, CH, CH1, CH2, CH3, CE1, CE2, CE3, CD1, CD2, CD3, CG1, CG2, CG3, CM, CM1, CM2, CM3, CA1, CA2, CA3, C1’, C2’, C3’, C4’, C5’, C2, C4, C5, C6, C8
Nitrogen	N, ND1, ND2, NE, NE1, NE2, NH1, NH2, NZ, N1, N2, N3, N6, N7, N9
Oxygen	O, OXT, OD1, OD2, OE1, OE2, OG, OG1, OG2, OH, OH1, OH2, O2’, O3’, O4’, O5’, O2P, O3P, O1P, O2, O4, O6
Phosphorus	P, OP1, OP2, OP3
Sulfur	SG, SD
Virtual ring centers	RING_3, RING_4, RING_5, RING_6, RING_X
Fallback	X (unknown protein atom)

Table S2. Ligand Atom Vocabulary

Category	Supported atom names
Main-group nonmetals	`C`, `O`, `N`, `F`, `S`, `P`
Halogens	`Cl`, `Br`, `I`

Category	Supported atom names
Transition metals	ZN, MG, FE, CU, MN, CO, NI, MO, W
Others	SE
Fallback	X (unknown ligand atom)

Table S3. Dataset Features.

Feature Name	Shape	Data Type	Description
x	[n, 3]	float32	3D coordinates of all atoms (receptor + ligand). Each row contains (x, y, z) coordinates in Angstrom.
h	[n, h_dim]	float32	Node features for each atom. Contains molecular type encoding (3 dimensions: backbone, side chain, ligand) and atom type one-hot encoding.
z	[n, n, 1]	float32	Pair features matrix. Contains is_same_residue feature: 1.0 if two receptor atoms belong to the same residue, 0.0 otherwise. Ligand atoms are assigned -1 residue index.
receptor_mask	[n]	int64	Binary mask indicating receptor atoms (1) vs ligand atoms (0).
seq_mask	[n]	int64	Binary mask indicating valid (non-padding) nodes. 1 for valid atoms, 0 for padding positions.
pair_mask	[n, n]	int64	Binary mask indicating valid (non-padding) pairs. 1 for valid atom pairs, 0 for pairs involving padding positions.

Notes

- n represents the number of atoms in a single sample (receptor + ligand).
- max_n represents the maximum number of atoms across all samples in a batch.
- h_dim represents the dimension of node features (molecular type encoding + atom type one-hot encoding).
- All features are padded to max_n in batched mode for efficient processing.

Table S4. Functional groups and SMARTS patterns

Functional Group	SMARTS Pattern
Alcohol	[CX4][OH]
Aldehyde	[CX3H1](=O)C
Amide	C(=O)N
Amine (Primary/Secondary)	[NX3;H2,H1;!$(NC=O)]
Amine (Tertiary)	[NX3]([#6])([#6])[#6]
Benzene	C1CCCCC1
Carboxylic Acid	C(=O)[OH]
Cyclobutane	C1CCC1
Cyclopropane	C1CC1
Epoxide	[C;R]1[O][C;R]1
Ester	C(=O)O*
Ether	[OD2]([#6])[#6]
Furan	C1CCCO1
Halogen	[F,Cl,Br,I]
Imidazole	C1CNC[NH]1
Indole	C1CC2CCCC2[NH]1
Ketone	C(=O)C
Nitrile	[C;!R]#[N]
Oxazole	C1COCNC1
Phenol	C1CCCCC1[OH]
Pyridine	N1CCCCC1
Pyrimidine	C1CNCNC1
Sulfonamide	S(=O)(=O)N
Thioether	[#16]([#6])[#6]
Thiol	[#16H1]
Thiophene	C1CCSC1

Supplementary Movie: Visualization of protein-ligand generation using a diffusion model.

REFERENCES

1. A. Vaswani, N. Shazeer, N. Parmar, J. Uszkoreit, L. Jones, A. N. Gomez, Ł. Kaiser, I. Polosukhin, Attention is all you need. *Adv. Neural Inf. Process. Syst.* **30**, (2017).

2. J. Ho, A. Jain, P. Abbeel, Denoising diffusion probabilistic models. *Adv. Neural Inf. Process. Syst.* **33**, 6840–6851 (2020).

3. D. D. Martinelli, Generative machine learning for de novo drug discovery: A systematic review. *Comput. Biol. Med.* **145**, 105403 (2022).

4. D. Weininger, SMILES, a chemical language and information system. 1. Introduction to methodology and encoding rules. *J. Chem. Inf. Comput. Sci.* **28**, 31–36 (1988).

5. M. Krenn, Q. Ai, S. Barthel, N. Carson, A. Frei, N. C. Frey, P. Friederich, T. Gaudin, A. A. Gayle, K. M. Jablonka, R. F. Lameiro, D. Lemm, A. Lo, S. M. Moosavi, J. M. Nápoles-Duarte, A. Nigam, R. Pollice, K. Rajan, U. Schatzschneider, P. Schwaller, M. Skreta, B. Smit, F. Strieth-Kalthoff, C. Sun, G. Tom, G. Falk von Rudorff, A. Wang, A. D. White, A. Young, R. Yu, A. Aspuru-Guzik, SELFIES and the future of molecular string representations. *Patterns* **3**, 100588 (2022).

6. M. Popova, M. Shvets, J. Oliva, O. Isayev, MolecularRNN: Generating realistic molecular graphs with optimized properties. arXiv:1905.13372 [cs.LG] (2019); https://doi.org/10.48550/arXiv.1905.13372.

7. Y. Li, L. Zhang, Z. Liu, Multi-objective de novo drug design with conditional graph generative model. *J. Chem.* **10**, 33–33 (2018).

8. F. Grisoni, M. Moret, R. Lingwood, G. Schneider, Bidirectional molecule generation with recurrent neural networks. *J. Chem. Inf. Model.* **60**, 1175–1183 (2020).

9. W. Jin, R. Barzilay, T. Jaakkola, "Junction tree variational autoencoder for molecular graph generation," in *Proceedings of the 35th International Conference on Machine Learning* (PMLR, 2018), pp. 2323–2332; https://proceedings.mlr.press/v80/jin18a.html).

10. M. Simonovsky, N. Komodakis, “GraphVAE: Towards generation of small graphs using variational autoencoders,” in *Artificial Neural Networks and Machine Learning – ICANN 2018*, V. Kůrková, Y. Manolopoulos, B. Hammer, L. Iliadis, I. Maglogiannis, Eds. (Springer International Publishing, 2018), vol. 11139, pp. 412–422; http://link.springer.com/10.1007/978-3-030-01418-6_41.

11. Q. Liu, M. Allamanis, M. Brockschmidt, A. Gaunt, Constrained graph variational autoencoders for molecule design. *Adv. Neural Inf. Process. Syst.* **31**, 7806–7815 (2018).

12. N. D. Cao, T. Kipf, MolGAN: An implicit generative model for small molecular graphs. arXiv:1805.11973 [stat.ML] (2022); https://doi.org/10.48550/arXiv.1805.11973.

13. K. Madhawa, K. Ishiguro, K. Nakago, M. Abe, GraphNVP: An invertible flow model for generating molecular graphs. arXiv:1905.11600 [stat.ML] (2019); https://doi.org/10.48550/arXiv.1905.11600.

14. C. Zang, F. Wang, “MoFlow: An invertible flow model for generating molecular graphs,” in *Proceedings of the 26th ACM SIGKDD International Conference on Knowledge Discovery & Data Mining* (ACM, 2020), pp. 617–626; https://dl.acm.org/doi/10.1145/3394486.3403104.

15. A. Schneuing, C. Harris, Y. Du, K. Didi, A. Jamasb, I. Igashov, W. Du, C. Gomes, T. L. Blundell, P. Lio, Structure-based drug design with equivariant diffusion models. *Nat. Comput. Sci.* **4**, 899–909 (2024).

16. V. G. Satorras, E. Hoogeboom, M. Welling, “E (n) equivariant graph neural networks,” in *International Conference on Machine Learning* (PMLR, 2021), pp. 9323–9332; https://proceedings.mlr.press/v139/satorras21a.html.

17. L. Huang, T. Xu, Y. Yu, P. Zhao, X. Chen, J. Han, Z. Xie, H. Li, W. Zhong, K.-C. Wong, H. Zhang, A dual diffusion model enables 3D molecule generation and lead optimization based on target pockets. *Nat. Commun.* **15**, 2657 (2024).

18. Z. Chen, B. Peng, T. Zhai, D. Adu-Ampratwum, X. Ning, Generating 3D small binding molecules using shape-conditioned diffusion models with guidance. *Nat. Mach. Intell.* **7**, 758–770 (2025).

19. H. Lin, Y. Huang, O. Zhang, S. Ma, M. Liu, X. Li, L. Wu, J. Wang, T. Hou, S. Z. Li, Diffbp: Generative diffusion of 3d molecules for target protein binding. *Chem. Sci.* **16**, 1417–1431 (2025).

20. J. Guan, W. W. Qian, X. Peng, Y. Su, J. Peng, J. Ma, 3D equivariant diffusion for target-aware molecule generation and affinity prediction. arXiv:2303.03543 [q-bio.BM] (2023); https://doi.org/10.48550/arXiv.2303.03543.

21. F. Ding, N. V. Dokholyan, Incorporating backbone flexibility in MedusaDock improves ligand-binding pose prediction in the CSAR2011 docking benchmark. *J. Chem. Inf. Model.* **53**, 1871–1879 (2013).

22. J. Wang, N. V. Dokholyan, MedusaDock 2.0: Efficient and accurate protein–ligand docking with constraints. *J. Chem. Inf. Model.* **59**, 2509–2515 (2019).

23. D. E. Koshland, The Key-lock theory and the induced fit theory. *Angew. Chem. Int. Ed. Engl.* **33**, 2375–2378 (1995).

24. N. V. Dokholyan, Controlling allosteric networks in proteins. *Chem. Rev.* **116**, 6463–6487 (2016).

25. J. Wang, N. V. Dokholyan, Leveraging transfer learning for predicting protein–small-molecule interaction predictions. *J. Chem. Inf. Model.* **65**, 3262–3269 (2025).

26. J. Wang, N. V. Dokholyan, Yuel: Improving the generalizability of structure-free compound-protein interaction prediction. *J. Chem. Inf. Model.* **62**, 463–471 (2022).

27. S. Yin, L. Biedermannova, J. Vondrasek, N. V. Dokholyan, MedusaScore: An accurate force-field based scoring function for virtual drug screening. *J. Chem. Inf. Model.* **48**, 1656–1662 (2008).

28. V. R. Chirasani, J. Wang, C. Sha, W. Raup-Konsavage, K. Vrana, N. V. Dokholyan, Whole proteome mapping of compound-protein interactions. *Curr. Res. Chem. Biol.* **2**, 100035 (2022).

29. K. A. Reynolds, R. N. McLaughlin, R. Ranganathan, Hot spots for allosteric regulation on protein surfaces. *Cell* **147**, 1564–1575 (2011).

30. J. Austin, D. D. Johnson, J. Ho, D. Tarlow, R. van den Berg, Structured denoising diffusion models in discrete state-spaces. arXiv:2107.03006 [cs.LG] (2023); https://doi.org/10.48550/arXiv.2107.03006.

31. E. Hoogeboom, V. G. Satorras, C. Vignac, M. Welling, Equivariant diffusion for molecule generation in 3D. arXiv:2203.17003 [cs.LG] (2022); https://doi.org/10.48550/arXiv.2203.17003.

32. G. R. Bickerton, G. V. Paolini, J. Besnard, S. Muresan, A. L. Hopkins, Quantifying the chemical beauty of drugs. *Nat. Chem.* **4**, 90–98 (2012).

33. P. Ertl, A. Schuffenhauer, Estimation of synthetic accessibility score of drug-like molecules based on molecular complexity and fragment contributions. *J. Cheminform* **1**, 8 (2009).

34. M. Swain, MolVS: Molecule Validation and Standardization [Computer software]. GitHub (2018); https://github.com/mcs07/MolVS.

35. J. Eberhardt, D. Santos-Martins, A. F. Tillack, S. Forli, AutoDock Vina 1.2.0: New docking methods, expanded force field, and Python bindings. *J. Chem. Inf. Model* **61**, 3891–3898 (2021).

36. G. Kontopidis, C. McInnes, S. R. Pandalaneni, I. McNae, D. Gibson, M. Mezna, M. Thomas, G. Wood, S. Wang, M. D. Walkinshaw, P. M. Fischer, Differential binding of inhibitors to active and inactive CDK2 provides insights for drug design. *Chem. Biol.* **13**, 201–211 (2006).

37. X.-J. Chu, W. DePinto, D. Bartkovitz, S.-S. So, B. T. Vu, K. Packman, C. Lukacs, Q. Ding, N. Jiang, K. Wang, P. Goelzer, X. Yin, M. A. Smith, B. X. Higgins, Y. Chen, Q. Xiang, J. Moliterni, G. Kaplan, B. Graves, A. Lovey, N. Fotouhi, Discovery of [4-amino-2-

(1-methanesulfonylpiperidin-4-ylamino)pyrimidin-5-yl] (2,3-difluoro-6- methoxyphenyl) methanone (R547), a potent and selective cyclin-dependent kinase inhibitor with significant in vivo antitumor activity. *J. Med. Chem.* **49**, 6549–6560 (2006).

38. L. Shewchuk, A. Hassell, B. Wisely, W. Rocque, W. Holmes, J. Veal, L. F. Kuyper, Binding mode of the 4-anilinoquinazoline class of protein kinase inhibitor: X-ray crystallographic studies of 4-anilinoquinazolines bound to cyclin-dependent kinase 2 and p38 kinase. *J. Med. Chem.* **43**, 133–138 (2000).

39. H. N. Bramson, J. Corona, S. T. Davis, S. H. Dickerson, M. Edelstein, S. V. Frye, R. T. Gampe, P. A. Harris, A. Hassell, W. D. Holmes, R. N. Hunter, K. E. Lackey, B. Lovejoy, M. J. Luzzio, V. Montana, W. J. Rocque, D. Rusnak, L. Shewchuk, J. M. Veal, D. H. Walker, L. F. Kuyper, Oxindole-based inhibitors of cyclin-dependent kinase 2 (CDK2): Design, synthesis, enzymatic activities, and x-ray crystallographic analysis. *J. Med. Chem.* **44**, 4339–4358 (2001).

40. C. Hamdouchi, H. Keyser, E. Collins, C. Jaramillo, J. E. De Diego, C. D. Spencer, J. A. Dempsey, B. D. Anderson, T. Leggett, N. B. Stamm, R. M. Schultz, S. A. Watkins, K. Cocke, S. Lemke, T. F. Burke, R. P. Beckmann, J. T. Dixon, T. M. Gurganus, N. B. Rankl, K. A. Houck, F. Zhang, M. Vieth, J. Espinosa, D. E. Timm, R. M. Campbell, B. K. R. Patel, H. B. Brooks, The discovery of a new structural class of cyclin-dependent kinase inhibitors, aminoimidazo[1,2-a]pyridines. *Mol. Cancer Ther.* **3**, 1–9 (2004).

41. M. Brylinski, J. Skolnick, What is the relationship between the global structures of apo and holo proteins? *Proteins* **70**, 363–377 (2008).

42. M. L. Benson, R. D. Smith, N. A. Khazanov, B. Dimcheff, J. Beaver, P. Dresslar, J. Nerothin, H. A. Carlson, Binding MOAD, a high-quality protein–ligand database. *Nucleic Acids Res.* **36**, D674–D678 (2007).

43. H. M. Berman, T. N. Bhat, P. E. Bourne, Z. Feng, G. Gilliland, H. Weissig, J. Westbrook, The Protein Data Bank and the challenge of structural genomics. *Nat. Struct. Mol. Biol.* **7**, 957–959 (2000).

44. G. M. Boratyn, C. Camacho, P. S. Cooper, G. Coulouris, A. Fong, N. Ma, T. L. Madden, W. T. Matten, S. D. McGinnis, Y. Merezhuk, Y. Raytselis, E. W. Sayers, T. Tao, J. Ye, I. Zaretskaya, BLAST: A more efficient report with usability improvements. *Nucleic Acids Res.* **41**, W29–W33 (2013).

45. Y. Zhang, J. Skolnick, TM-align: A protein structure alignment algorithm based on the TM-score. *Nucleic Acids Res.* **33**, 2302–2309 (2005).

46. E. S. R. Ehmki, R. Schmidt, F. Ohm, M. Rarey, Comparing molecular patterns using the example of SMARTS: Applications and filter collection analysis. *J. Chem. Inf. Model.* **59**, 2572–2586 (2019).

47. L. L. C. Schrodinger, The PyMOL molecular graphics system. *Version* **1**, 0–0 (2010).

48. N. M. O'Boyle, M. Banck, C. A. James, C. Morley, T. Vandermeersch, G. R. Hutchison, OpenBabel: An open chemical toolbox. *J. Cheminformatics* **3**, 33 (2011).
